# Ceftazidime and Usnic Acid Encapsulated in Chitosan-Coated Liposomes for Oral Administration against Colorectal Cancer-Inducing *Escherichia coli*

**DOI:** 10.3390/ph17060802

**Published:** 2024-06-19

**Authors:** Jaqueline Barbosa de Souza, Davi de Lacerda Coriolano, Rayza Camila dos Santos Silva, Sérgio Dias da Costa Júnior, Luís André de Almeida Campos, Iago Dillion Lima Cavalcanti, Mariane Cajubá de Britto Lira Nogueira, Valéria Rêgo Alves Pereira, Maria Carolina Accioly Brelaz-de-Castro, Isabella Macário Ferro Cavalcanti

**Affiliations:** 1 Institute Keizo Asami (iLIKA), Federal University of Pernambuco (UFPE), Recife 50670-901, PE, Brazil; jaqueline.bsouza@ufpe.br (J.B.d.S.); davi.coriolano@ufpe.br (D.d.L.C.); rayza.silva@aluno.uepb.edu.br (R.C.d.S.S.); sergio_dias16@hotmail.com (S.D.d.C.J.); luis.andre@ufpe.br (L.A.d.A.C.); iago.dillion@ufpe.br (I.D.L.C.); mariane.lira@ufpe.br (M.C.d.B.L.N.); 2 Laboratory of Nanotechnology, Biotechnology and Cell Culture (NanoBioCel), Academic Center of Vitória (CAV), Federal University of Pernambuco (UFPE), Vitória de Santo Antão 55608-680, PE, Brazil; 3 Department of Immunology, Aggeu Magalhães Institute (IAM/FIOCRUZ), Federal University of Pernambuco (UFPE), Recife 50670-420, PE, Brazil; valeriaph@gmail.com; 4 Laboratory of Parasitology, Academic Center of Vitoria (CAV), Federal University of Pernambuco (UFPE), Vitória de Santo Antão 55608-680, PE, Brazil; 5 Laboratory of Microbiology and Immunology, Academic Center of Vitória (CAV), Federal University of Pernambuco (UFPE), Vitória de Santo Antão 55608-680, PE, Brazil

**Keywords:** lipid nanocarriers, polymers, Enterobacteriaceae, carcinogenesis

## Abstract

*Escherichia coli* has been associated with the induction of colorectal cancer (CRC). Thus, combined therapy incorporating usnic acid (UA) and antibiotics such as ceftazidime (CAZ), co-encapsulated in liposomes, could be an alternative. Coating the liposomes with chitosan (Chi) could facilitate the oral administration of this nanocarrier. Liposomes were prepared using the lipid film hydration method, followed by sonication and chitosan coating via the drip technique. Characterization included particle size, polydispersity index, zeta potential, pH, encapsulation efficiency, and physicochemical analyses. The minimum inhibitory concentration and minimum bactericidal concentration were determined against *E. coli* ATCC 25922, NCTC 13846, and H10407 using the microdilution method. Antibiofilm assays were conducted using the crystal violet method. The liposomes exhibited sizes ranging from 116.5 ± 5.3 to 240.3 ± 3.5 nm and zeta potentials between +16.4 ± 0.6 and +28 ± 0.8 mV. The encapsulation efficiencies were 51.5 ± 0.2% for CAZ and 99.94 ± 0.1% for UA. Lipo-CAZ-Chi and Lipo-UA-Chi exhibited antibacterial activity, inhibited biofilm formation, and preformed biofilms of *E. coli*. The Lipo-CAZ-UA-Chi and Lipo-CAZ-Chi + Lipo-UA-Chi formulations showed enhanced activities, potentially due to co-encapsulation or combination effects. These findings suggest potential for in vivo oral administration in future antibacterial and antibiofilm therapies against CRC-inducing bacteria.

## 1. Introduction

Colon and rectum cancer, also known as bowel or colorectal cancer (CRC), represents approximately 10.2% of cancer cases worldwide. According to estimates by the International Agency for Research on Cancer, mortality rates are approximated at 881,000 deaths per year [1,2].

Gut microbiota bacteria may be related to the development of carcinogenesis. In this sense, *Escherichia coli* is frequently isolated from patients with CRC and healthy individuals; however, more pathogenic strains are found in patients with CRC compared to healthy ones [3,4]. There is evidence that bacteria, such as genotoxic strains of *E. coli*, can induce a specific cytopathic effect known as megalocytosis. This megalocytosis can cause chromosomal instability and cell cycle arrest, accompanied by the secretion of inflammatory mediators and growth factors, thus promoting cell proliferation, factors that significantly contribute to CRC carcinogenesis [5,6,7].

Thus, it is necessary to develop therapeutic strategies to prevent the initiation and progression of CRC caused by Gram-negative bacteria, with the use of antibiotics being a proposed solution with potential applications [8]. Ceftazidime (CAZ), a third-generation cephalosporin, has a spectrum of actions against *E. coli*; however, due to its cationic characteristic and high polarity, its absorption is reduced by the gastrointestinal tract, resulting in low bioavailability when administered orally [9,10].

Combined therapies represent an alternative in the treatment of bacterial diseases, being used to increase potency and, consequently, therapeutic success. Natural products have the potential to act in synergy with drugs conventionally used in therapy, thus reducing the dose needed to achieve the desired effect and causing a reduction in side effects [11,12].

In this context, usnic acid (UA) represents a potential compound for this purpose. UA is a product derived from the secondary metabolism of lichens, presenting several pharmacological activities such as antibacterial, antiviral, antifungal, anti-inflammatory, and analgesic, as well as the potential inhibition of bacterial biofilms [13,14]. However, although promising, UA has low aqueous solubility and forms crystalline aggregates, in addition to hepatotoxicity, making its clinical application difficult [15,16].

Therefore, it is necessary to develop an alternative that reduces the toxic potential of CAZ and UA and improves their pharmacological properties [17]. A viable alternative is the use of controlled-release systems (CRSs), as these systems enable an efficient and safe therapy for drug administration with a reduction in side effects.

Among the nanosystems used as CRSs, liposomes stand out [18]. Due to their amphiphilic nature, the encapsulation of drugs in liposomes protects them from physiological degradation [19,20,21]. However, liposomes are sensitive to stomach pH. Chitosan is a polysaccharide with cationic properties, used due to its bioadhesion, biocompatibility, and biodegradability; therefore, it can be used as a coating molecule for oral administration [22,23]. Thus, the coating of a liposome with chitosan can improve the absorption of a drug in the intestine administered orally, in addition to increasing the distribution of the drug in bacterial cells [24,25,26,27,28].

Therefore, the aim of this work was to develop, characterize, and evaluate the antibacterial activity of CAZ and UA encapsulated in chitosan-coated liposomes against CRC-inducing bacteria. Thus, it is suggested that the system proposed by the present study can be used in patients with a positive biopsy for CRC in the presence of positive *E. coli* pKs as oral pre-antineoplastic therapy. The use of this therapeutic strategy may provide a necessary therapeutic innovation for the prevention of CRC. As far as we know, this is the first study to carry out the encapsulation of CAZ and UA in chitosan-coated liposomes, thus being a pioneer in the area.

## 2. Results and Discussion

### 2.1. Characterization of the Liposomes

#### 2.1.1. Particle Size, Polydispersity Index, Zeta Potential, pH, and Encapsulation Efficiency of the Liposomes

The liposomes were characterized by their Ø, PDI, ζ, pH, and %EE (Table 1).

**Figure 1:**
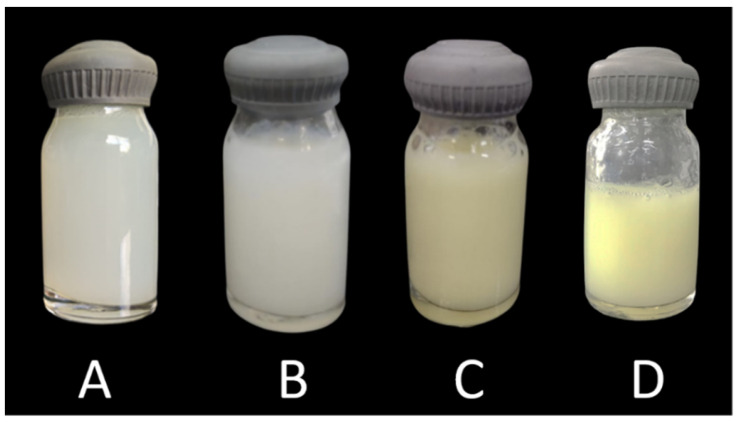
Macroscopic appearance of CHI-coated liposomes (Lipo-Chi) (**A**), CHI-coated liposomes encapsulating CAZ (Lipo-CAZ-Chi) (**B**), CHI-coated liposomes encapsulating UA (Lipo-UA-Chi) (**C**), and CHI-coated liposomes co-encapsulating CAZ and UA (Lipo-CAZ-UA-Chi) (**D**).

As shown in Figure 1, the macroscopic appearance of the Lipo-Chi (Figure 1A) and Lipo-CAZ-Chi (Figure 1B) formulations was uniform, without flocculation, with a milky white color and light blue opalescence. Lipo-UA-Chi (Figure 1C) and Lipo-CAZ-UA-Chi (Figure 1D) had the same characteristics as mentioned above, but the color of these formulations was yellowish due to the presence of UA.

All the formulations exhibited characteristics consistent with nanometer-scale particles, showing no signs of precipitation or phase separation. In addition, the particle size was compatible with possible oral administration, which leads to greater acceptance by patients when compared to the parenteral route [29,30,31,32]. As for the zeta potential, all the formulations showed a positive surface charge, favoring stronger electrostatic repulsion between particles, leading to better stability in these nanocarriers [33,34,35,36,37].

Additionally, Chi’s mucoadhesive capacity facilitates its oral administration, as it adheres to the intestinal wall, promoting increased drug contact with the organ and enhancing absorption by intestinal epithelial cells, thus improving bioavailability [38,39]. The acidic pH of the liposomes, stemming from Chi’s cationic nature [40], further aids absorption in the intestine, as acidic formulations are more readily absorbed [41,42,43]. Moreover, when coated with polysaccharides like Chi, pH plays a role in drug release, with the formulations engineered to withstand gastric pH and release in the small intestine [44].

The challenges associated with non-parenteral drug administration, particularly orally, are significant in the pharmaceutical industry due to issues such as low oral availability, physiological inactivation, and poor permeability through the gastrointestinal tract epithelium. To address these challenges, pharmaceutical nanotechnology, including the use of liposomes coated with polysaccharides like Chi, has emerged as a promising alternative [45,46,47].

Previous studies have encapsulated CAZ in conventional liposomes without Chi coating, achieving encapsulation efficiencies ranging from 5.77% to 50% [47,48,49]. Ceftazidime’s hydrophilic nature (log P: −1.6) prevents its entrapment within the lipid bilayer, directing it instead to the aqueous compartment of the liposome or dispersing it in the external aqueous medium [48,50]. A comparison of CAZ’s encapsulation efficiency in the Chi-coated liposomes developed in this study with findings from the literature suggests promising results, as Chi’s interaction with the drug potentially enhances its encapsulation capacity [29,51,52,53].

The high %EE of UA in Lipo-UA-Chi highlights the efficacy of the encapsulation method used in this study. Furthermore, UA’s low water solubility, attributed to its molecular structure and the formation of hydrogen-type intermolecular bonds, facilitates its inclusion in the lipid bilayer, streamlining the encapsulation process [15,16]. These results are consistent with prior studies on UA encapsulation, where a UA content exceeding 90% and a %EE close to 99% have been reported [54,55,56].

Maintaining an adequate drug concentration at the site of action is crucial for treating infections induced by bacteria associated with colorectal cancer [38]. Hence, formulations with a high drug content and encapsulation efficiency are essential for effective pharmacotherapy [53,57]. However, achieving optimal %EE can be challenging, as it depends on factors like liposome properties and drug interactions. Co-encapsulation may lead to drug agglomeration within liposomes, affecting the release dynamics and potentially causing repulsion between drugs during encapsulation [28,47,48,58]. Combining antimicrobial agents is a strategy that has already been used for decades in clinical practice to increase efficacy, whether by expanding the antimicrobial spectrum, reducing toxicity, or even preventing the emergence of resistant strains [59]. In this sense, using liposomes as carriers of antibiotics containing both drugs, either in the form of co-encapsulation or in the form of a mixture (*pool*), can favor their controlled distribution in the affected tissues, strengthening their synergy [49,60].

#### 2.1.2. FTIR and XRD of the Liposomes

**Figure 2:**
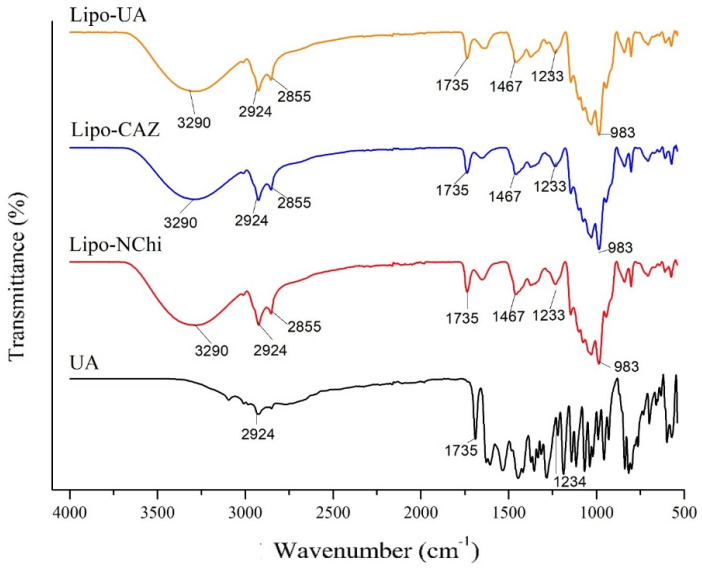
FTIR spectra of uncoated CHI liposomes (Lipo-UA, Lipo-CAZ, and Lipo-NChi) and UA.

FTIR of the formulations Lipo-UA, Lipo-CAZ, non-chitosan-coated liposomes (Lipo-NChi), and UA was carried out to assess the presence of critical functional groups capable of portraying the presence of bands characteristic of UA, CAZ, and lipid content (Figure 2).

Considering that FTIR is a fundamental analysis technique for evaluating the presence of functional groups that can indicate the presence of the compounds present in liposomes, in the FTIR spectra of all the liposomal formulations without the CHI coating, a band was verified at 2855 and 3290 cm^−1^, which indicated the presence of hydrogen bonds, which represent the main macromolecular interactions of, for example, phospholipids, corresponding to the symmetric stretching CH_2_ and O-H, respectively [61]. The bands at 1233 and 1735 cm^−1^, in turn, corresponded to the phosphate (P=O) and ester (C=O) groups, both present at the polar end of the phospholipid structure, and at 2924 and 2854 cm^−1^, indicating the presence of their hydrophobic chain [62].

In the Lipo-CAZ spectrum, the axial strain bands in the regions between 3290 and 2924 cm^−1^ corresponded to the chemical groups OH and NH, characteristic of cephalosporin compounds; the axial strain at 1367 cm^−1^ indicates the presence of C-N bonds of aromatic rings, and that at 1736 cm^−1^ refers to the amide group [63]. The C-H and C=O bonds present in the β-lactam ring are visible at 2924 and 1753 cm^−1^. Furthermore, a change in peak intensity was observed between 1736 and 1367 cm^−1^, indicating that there was an interaction between the liposome and CAZ [64].

The UA spectrum showed characteristic peaks at 1690 and 1067 cm^−1^, as previously reported in the literature [65]. Possible intramolecular hydrogen bonds contributed to the shortest wavelength position of aromatic methylketone at 1627 cm^−1^. The vibrations of the conjugated cyclic ketone group were seen at 1690 cm^−1^, in addition to antisymmetric bands at 1187 cm^−1^ corresponding to C-O-C bonds [66]. In the Lipo-UA spectrum, bands like those of UA were observed, indicating that its incorporation into the liposome did not change the stability of the drug. Furthermore, changes in the intensity and shape of the peaks at 1735 and 1234 cm^−1^ indicated the interaction between UA and the liposome [54].

**Figure 3:**
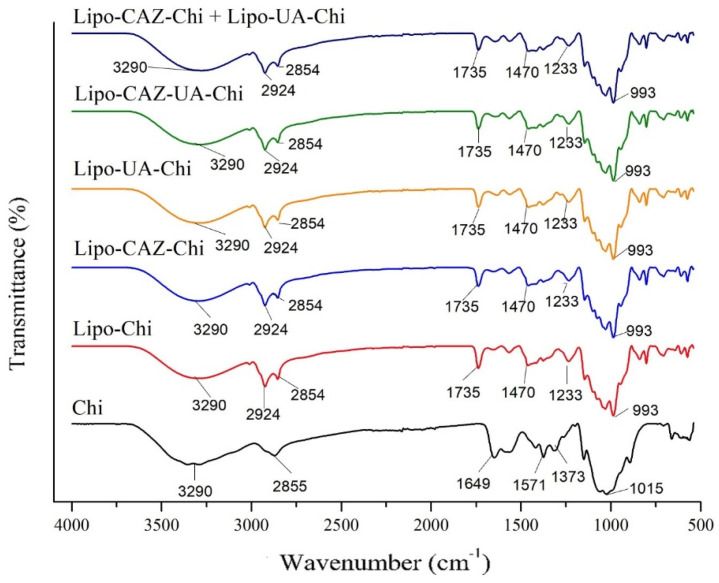
FTIR spectra of liposomes coated with Chi (Lipo-Chi, Lipo-UA-Chi, Lipo-CAZ-Chi, Lipo-CAZ-UA-Chi, and Lipo-CAZ-Chi + Lipo-UA-Chi) and CHI.

Figure 3 shows the FTIR spectra of the formulations with the CHI coating, namely Lipo-Chi, Lipo-CAZ-Chi, Lipo-UA-Chi, Lipo-CAZ-UA-Chi, Lipo-CAZ-Chi + Lipo-UA-Chi, and free CHI.

After coating the liposomes with Chi (Lipo-Chi), the characteristic lipid peak at 1735 cm^−1^ changed to lower frequency values (1733 cm^−1^), indicating the possible formation of new hydrogen bonds [67]. The interaction between the phosphate group present in phospholipids and Chi resulted in the removal of water from it, shifting the band to a higher frequency at 1981 cm^−1^. N-O stretching vibrations were apparent at 3355 cm^−1^ in the Chi spectrum, which shifted to a lower frequency (3289 cm^−1^) in the spectra of the Chi-coated liposomes [68]. The N-H bending band in the Chi spectrum (1575 cm^−1^) was modified to 1566 cm^−1^ in the Lipo-Chi spectrum, suggesting an electrostatic interaction between the amine groups of Chi and the liposome phosphates. These data corroborate the study by Hamedinasab et al. [69], who also observed these bands when coating liposomes containing N-acetylcysteine with Chi.

In the Lipo-CAZ-Chi spectrum, a band was observed at 3011–3292 cm^−1^, referring to the groups =C-H, N-H, and O-H, in addition to an elongation at 1736 cm^−1^, referring to the presence of a bond between C=O, this being attributed to the association of this group with the group present in the polar head of the lipid bilayer. Another elongation was visualized at 1566 cm^−1^, referring to the axial deformation of the C=C group; an axial deformation was also formed at 1100 cm^−1^ by C-O and at 609–803 cm^−1^ by hydrogen deformations [64]. In the spectra of Lipo-CAZ-UA-Chi and Lipo-CAZ-Chi + Lipo-UA-Chi, the displacement of the bands from 1566 to 1564 cm^−1^ (Lipo-CAZ-UA-Chi), 1736 to 1735 cm^−1^, and 1566 to 1562 cm^−1^ (Lipo-CAZ-Chi + Lipo-UA-Chi) was evident. Despite these displacements, the spectra were like that of Lipo-Chi, suggesting that CAZ was successfully encapsulated in the hydrophilic region of the liposomes [70,71].

The Lipo-UA-Chi spectrum, in turn, showed characteristic bands of the presence of UA at 1637 and 1075 cm^−1^, which are equivalent to the C=O vibrations of amides and axial deformations of C-O-type esters. Furthermore, the axial strain vibrations at 2924 cm^−1^ due to primary or secondary C-H vibrations and the angular strains at 1033 to 1146 cm^−1^ are equivalent to the C-O bonds of primary and tertiary alcohol, respectively. It was also observed that there was a band shift in Lipo-CAZ-UA-Chi from 1637 to 1642 cm^−1^ and 1100 to 1099 cm^−1^, as well as a shift from 1637 to 1644 cm^−1^ for Lipo-CAZ-Chi + Lipo-UA-Chi [72].

Thus, in Figure 3, it is possible to observe that in all the liposomes, characteristic absorption bands of Chi and lipids are evident; however, in the formulations containing UA and CAZ, band shifts are observed, suggesting interactions with Chi and the effective encapsulation of these molecules.

**Figure 4:**
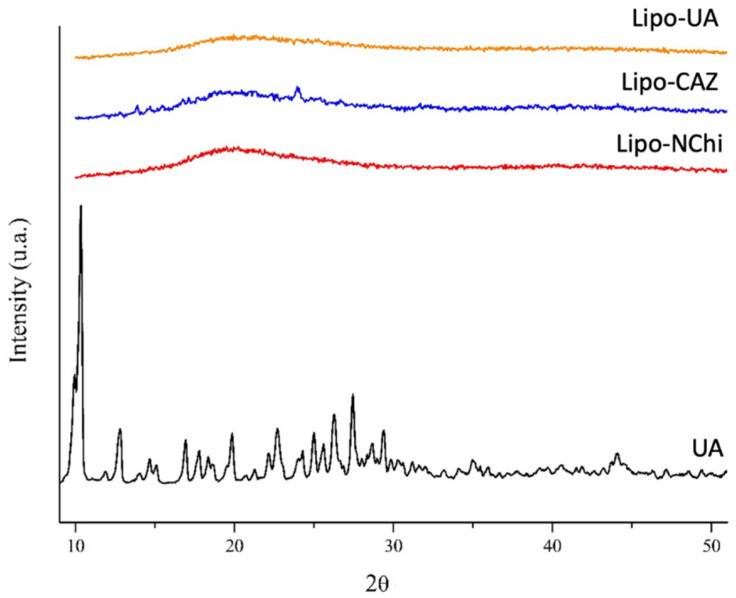
XRD of liposomes without drug or Chi (Lipo-NChi), with UA (Lipo-UA), and with CAZ (Lipo-CAZ) and free UA.

XRD studies were carried out to investigate the microstructure of the drugs, phospholipids, and Chi and their interactions with the liposomes developed. Figure 4 shows the XRD for the formulations without the Chi coating (Lipo-UA, Lipo-CAZ, Lipo-NChi) and UA.

As expected, pure UA exhibited all the characteristic peaks expected for UA crystals, which were in the range of different 2ϴ values, such as 10.3°, 14.5°, 18.6°, 22.7°, 24.3°, and 27.4° [54,73]. The Lipo-NChi diffractogram exhibited only a single broad peak, with a maximum value of around 2θ = 21°. However, after UA encapsulation in the liposome, the XRD patterns changed considerably compared to that of free UA, which indicates the amorphous nature of the sample, which is explained by the formation of a system with less organization along with the formation of molecular interactions between the UA and the liposome [31,54].

Lipo-CAZ, in turn, demonstrated a peak at 2θ = 25°; however, this does not correspond to that previously described in the literature for CAZ (2θ of 20.2°, 21.5°, and 22.3°), indicating the degree of crystallinity of the liposomes, as well as possible hydrogen bonds or electrostatic interactions between the drug and the lipids [74].

The Lipo-NChi diffractogram showed only a single broad peak, with a maximum value of around 2θ = 21°. However, after encapsulating UA in the liposomes, the XRD patterns changed considerably compared to that of free UA. Lipo-CAZ, on the other hand, showed a peak at 2θ = 25°.

**Figure 5:**
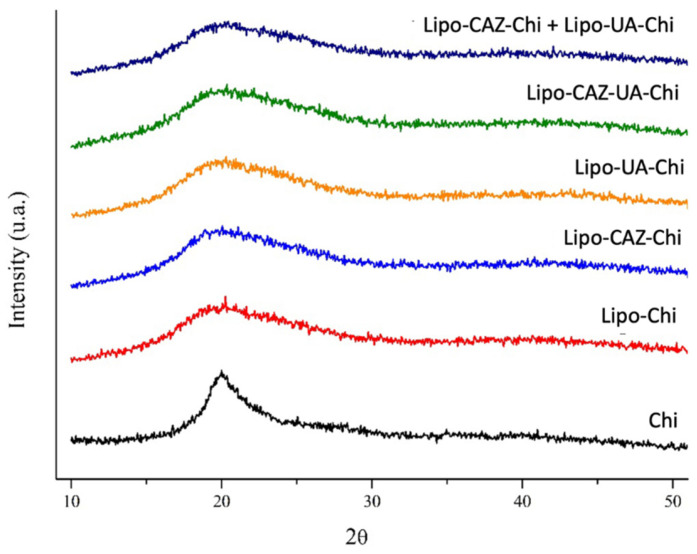
XRD of Chi-coated liposomes (Lipo-Chi, Lipo-UA-Chi, Lipo-CAZ-Chi, Lipo-CAZ-UA-Chi, Lipo-CAZ-Chi + Lipo-UA-Chi), and Chi.

Figure 5 shows the XRD of the formulations with the Chi coating (Lipo-Chi, Lipo-UA-Chi, Lipo-CAZ-Chi, Lipo-CAZ-UA-Chi, Lipo-CAZ-Chi + Lipo-UA-Chi), as well as Chi.

Chi showed peaks corresponding to those found in previous studies, such as the low-intensity peak around 2θ = 20° observed in the Lipo-Chi diffractogram, corresponding to crystalline forms of type α and β, which indicate the typical characteristics of this polymer in a semicrystalline state. On the other hand, the other diffractograms consisted of a superimposition of the individual patterns of the main components, with a masking of the peaks that characterize CAZ and UA [73,75].

The Lipo-Chi diffractogram exhibited a low-intensity peak around 2θ = 20°, indicating the presence of chemical interactions between the components and Chi. Nonetheless, the other diffractograms consisted of an overlapping of the individual patterns of the main components, phospholipids and Chi, with an occultation of the peaks that characterize CAZ and UA, suggesting their amorphization/dispersion in the liposome [76,77].

#### 2.1.3. Thermal Analysis through Thermogravimetry Analysis and Differential Scanning Calorimetry of the Liposomes

**Figure 6:**
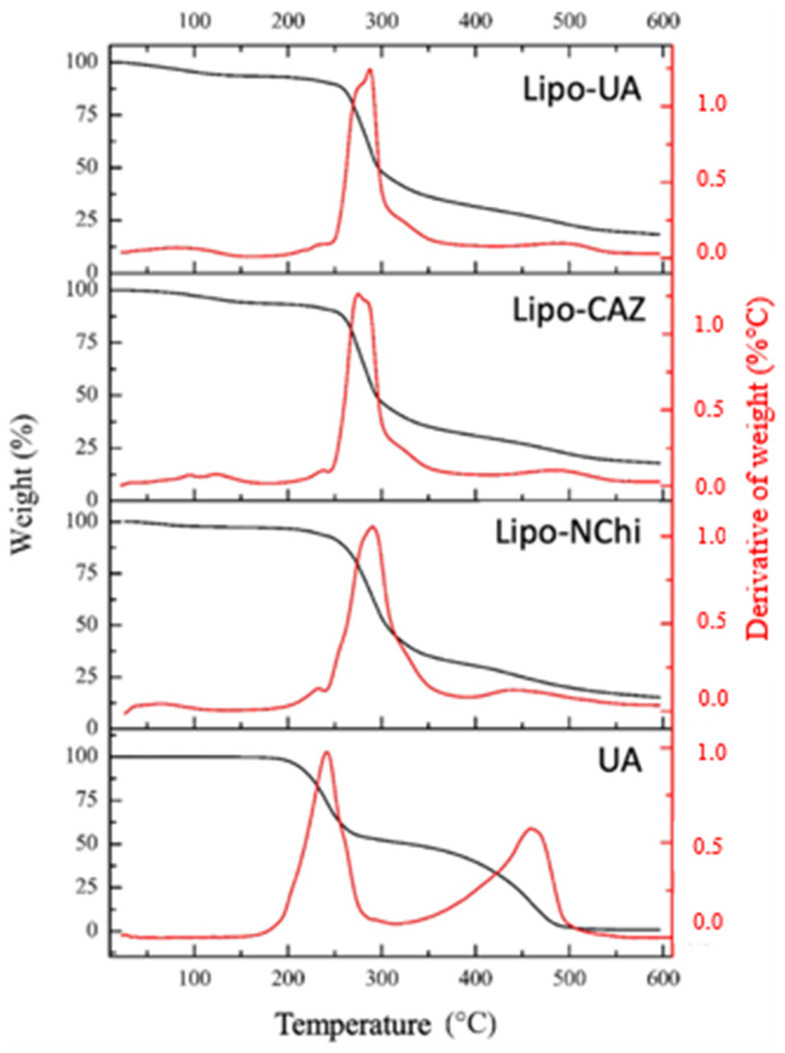
Thermogram of liposomes without Chi (Lipo-NChi), with UA (Lipo-UA), and with CAZ (Lipo-CAZ) and free UA.

Figure 6 shows the thermogravimetric curves of Lipo-NChi, Lipo-CAZ, Lipo-UA, and UA. In Lipo-NChi, the first stage showed minimal mass loss (2.6%) in the temperature range of 25 to 232 °C; on the other hand, the second stage occurred between 200 and 290 °C, where there was a more significant mass loss in the samples (82.2%), and the third stage occurred at temperatures above 300 °C.

As for the thermal analyses, in the Lipo-NChi thermogravimetric curve (Figure 6), the first stage was characterized by the presence of residual water in the samples and low-molecular-weight substances, while the second stage referred to the degradation of lipids. The third step was related to the further decomposition of the formulations’ constituents [78].

According to the literature, CAZ presents an average mass loss of 10% at temperature intervals between 50 and 100 °C, and about a 75% mass loss occurs at temperatures above 190 °C [79]. UA, in turn, according to its thermogram, has a mass loss of 48.8% at temperatures between 25 and 241 °C and about 50.4% at temperatures above 459 °C. Thus, it can be noted that liposomal formulations exhibit better thermal stability when compared to free drugs [80].

**Figure 7:**
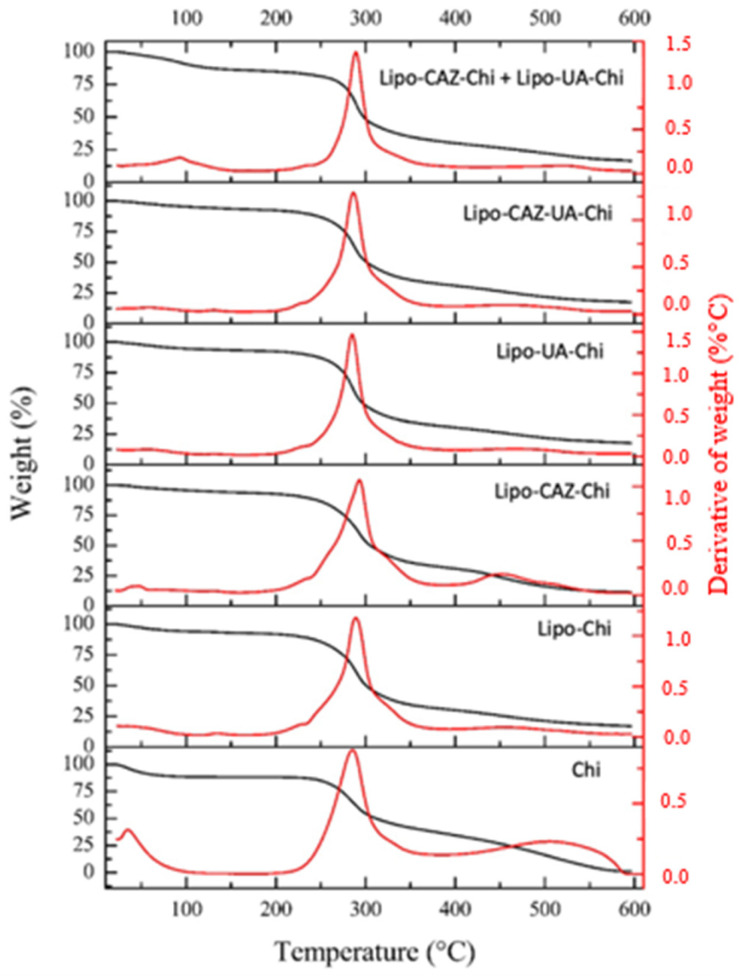
Thermograms of Chi-coated liposomes (Lipo-Chi, Lipo-UA-Chi, Lipo-CAZ-Chi, Lipo-CAZ-UA-Chi, Lipo-CAZ-Chi + Lipo-UA-Chi) and Chi.

Figure 7 shows the thermograms of the formulations with the Chi coating (Lipo-Chi, Lipo-UA-Chi, Lipo-CAZ-Chi, Lipo-CAZ-UA-Chi, Lipo-CAZ-Chi + Lipo-UA-Chi), as well as that of Chi. The thermogravimetric curves obtained from these liposomes showed an initial mass loss up to 226 °C of around 7% for Lipo-Chi, 6.1% for Lipo-CAZ-Chi, 6.5% for Lipo-UA-Chi, 6.8% for Lipo-CAZ-UA-Chi, and 14.1% for Lipo-CAZ-Chi + Lipo-UA-Chi, and a loss of ≥62.2% at temperatures above 285 °C. Chi had an initial loss of 11.62% at 35 °C, a further loss of 87% at 285 °C in the second phase, and finally a loss of 1.1% above 514 °C.

In Figure 7, the thermogravimetric curves obtained from these liposomes demonstrate a mass loss that was previously described in the literature and showed that there was no significant mass loss at temperatures between 25 and 226 °C, evidencing the efficient encapsulation of CAZ and UA [81,82].

**Figure 8:**
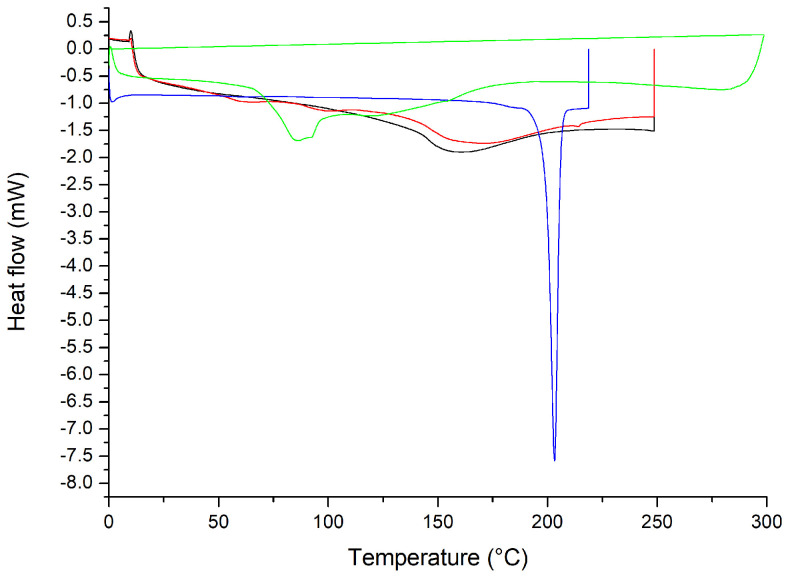
DSC of liposomes without chitosan: Lipo-NChi (**green line**), Lipo-UA (**black line**), Lipo-CAZ (**red line**), and free UA (**blue line**).

In the DSC thermograms, shown in Figure 8, of the formulations without Chi, the liposomes showed endothermic peaks: Lipo-NChi at 86.4, 94.4, and 128.5 °C; Lipo-CAZ at 86.1 and 159.1 °C; and Lipo-UA at 117.4 and 144.7 °C. Meanwhile, the free UA showed peaks at 199.4 and 202.9 °C.

In the DSC thermogram shown in Figure 8, the liposomes showed endothermic peaks. According to the literature, CAZ has a peak at 120 °C, while UA has melting peaks at 201.5, 204, and 273 °C [54,79]. In this sense, it is observed that the peaks corresponding to the degradation of drugs were not evidenced, suggesting that the CAZ and UA encapsulated in liposomes had improved thermal stability [80].

**Figure 9:**
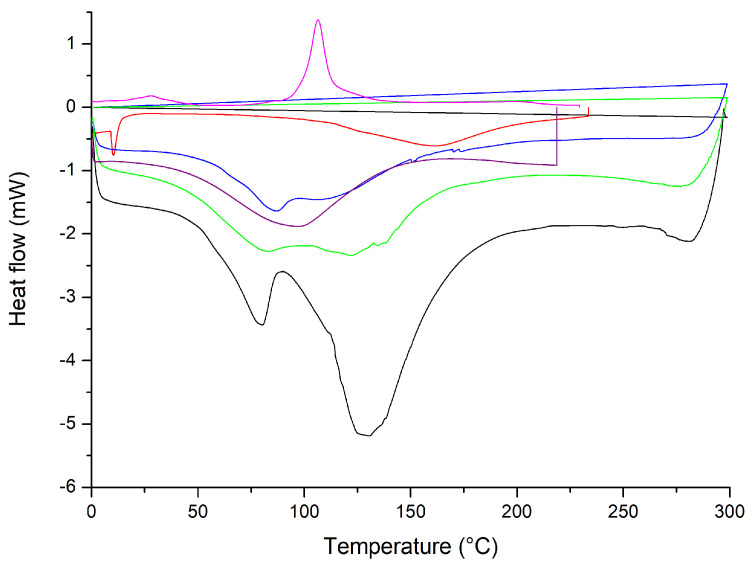
DSC of Chi-coated liposomes: Lipo-Chi (**black line**), Lipo-UA-Chi (**red line**), Lipo-CAZ-Chi (**blue line**), Lipo-CAZ-UA-Chi (**green line**), Lipo-CAZ-Chi + Lipo-UA-Chi (**pink line**), and Chi (**purple line**).

Figure 9 shows the DSC of the formulations with the chitosan coating (Lipo-Chi, Lipo-UA-Chi, Lipo-CAZ-Chi, Lipo-CAZ-UA-Chi, Lipo-CAZ-Chi + Lipo-UA-Chi), in addition to that of Chi. The endothermic peaks for Lipo-Chi were seen at 77.6 and 131.18 °C, Lipo-CAZ-Chi at 86.1 and 106.4 °C, Lipo-UA at 151 °C, Lipo-CAZ-UA-Chi at 83.1 and 121.9 °C, and Lipo-CAZ-Chi + Lipo-UA-Chi at 150 °C.

Figure 9 demonstrates the DSC of the formulations with the Chi coating (Lipo-Chi, Lipo-UA-Chi, Lipo-CAZ-Chi, Lipo-CAZ-UA-Chi, Lipo-CAZ-Chi + Lipo-UA-Chi), in addition to that of CHI. Considering the analysis of these formulations, the first peaks are usually related to the initial loss of water, attributing this to the presence of hydrophilic groups in both the lipids and the CHI. Furthermore, the characteristic peaks of CAZ and UA were not present in the thermogram, suggesting an interaction between them and the structure of the liposome. In this sense, it was possible to verify an improvement in the thermal stability of the formulations when compared to the formulations without the coating with Chi since changes in the melting temperature were evidenced due to the protection exerted by Chi against thermal degradation for the drugs [82].

#### 2.1.4. Stability of the Liposomes under Simulated Gastrointestinal pH Conditions

**Figure 10:**
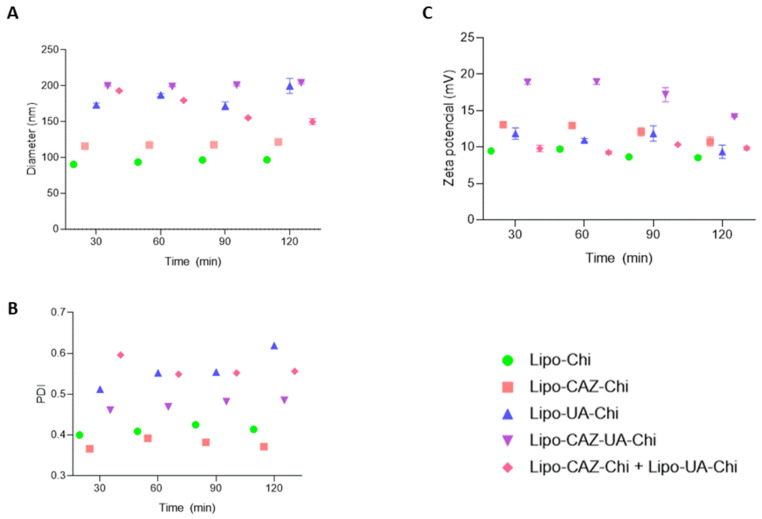
Average diameter (**A**), PDI (**B**), and zeta potential (**C**) of Lipo-Chi, Lipo-CAZ-Chi, Lipo-UA-Chi, Lipo-CAZ-UA-Chi, and Lipo-CAZ-Chi + Lipo-UA-Chi dispersions at pH 1.2.

**Figure 11:**
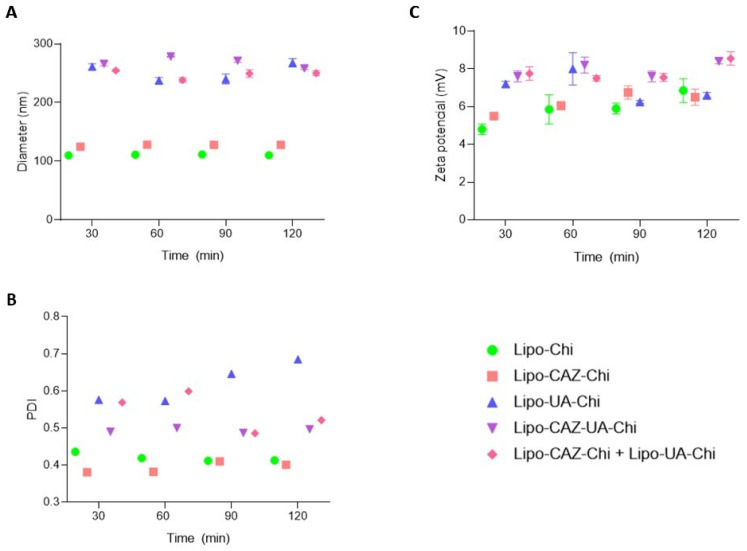
Average diameter (**A**), PDI (**B**), and zeta potential (**C**) of Lipo-Chi, Lipo-CAZ-Chi, Lipo-UA-Chi, Lipo-CAZ-UA-Chi, and Lipo-CAZ-Chi + Lipo-UA-Chi dispersions at pH 6.8.

Given the intended future oral administration of the liposomes developed in this study, it is vital to explore how pH affects their physicochemical properties for stability assessment. As illustrated in Figure 10A and Figure 11A, a gradual variation in the mean diameter of the liposomes was observed over a 120 min period at pH levels of 1.2 and 6.8. Subsequently, at pH 1.2, there was an approximate 3.8 nm decrease for all formulations, while at pH 6.8, an increase of around 0.1 nm was noted. Similar differences were observed in the PDI values, which increased from 0.2 to 0.1 after exposure to both pH 1.2 and 6.8. Following exposure to simulated biological fluids, the zeta potential gradually decreased at both pH 1.2 and 6.8, reducing by approximately 3 mV and 2 mV, respectively.

Considering the results shown in Figure 10, all the liposomes were stable during exposure to pH 1.2, which mimics the gastric pH, in the time interval of 30 to 120 min. Therefore, it was noted that the formulations underwent a small reduction in particle size and a small increase in PDI, attributed to the fact that Chi presents solubility in acidic conditions. The positive charge in Chi at pHs below its pKa (pH 6.5) makes its amino groups charged (–NH3+), and with that, there is a reduction in its internal osmolarity compared to the external environment, resulting in differences in osmotic pressure that may lead to the leakage of water present in the core of the liposomes to the external phase. This induces shrinkage of the liposomal membrane and a decrease in its size, as well as increasing the polydispersion between particles [83,84].

To provide further information about the mechanism of liposome stability, the surface charge was also determined at pH 1.2. Thus, a reduction in this parameter was also noted, possibly due to the partial release of Chi from the liposomal surface under acidic conditions, which discreetly reduces the zeta potential values [85,86]. Therefore, since the liposomes developed in this study proved to be stable under acidic conditions, they can withstand the acidic condition of the stomach and prevent the release of the encapsulated drug in this region.

After exposure to the simulated intestinal pH (pH 6.8) (Figure 11), there was an increase in the size of the liposome particles, as well as a reduction in the zeta potential, possibly due to the solution being above the pKa of the Chi amino groups, thus leading to deprotonation of NH3+ groups, leading to an increase in the colloidal aggregation of the particles, which, in turn, results in an increase in diameter and a reduction in surface charge [87,88].

Other studies have shown these same changes in these parameters after Chi-coated liposomes were exposed to simulated pHs of the gastrointestinal tract, as Zhou et al. [84] showed with the encapsulation of curcumin in Chi-coated liposomes for nutraceutical purposes and Hui and Huang [86] showed with Chi-coated liposomes for transporting bovine serum albumin in oral protein administration. Therefore, Lipo-CAZ-Chi, Lipo-UA-Chi, Lipo-CAZ-UA-Chi, and Lipo-CAZ-Chi + Lipo-UA-Chi demonstrate stability under simulated gastrointestinal conditions, enabling their application as intestinal antimicrobial oral delivery systems.

#### 2.1.5. Stability of the Liposomal Dispersions

The Ø, PDI, ζ, pH, drug content, and encapsulation efficiency of Lipo-CAZ-Chi (Table 2), Lipo-CAZ-UA-Chi (Table 3), and Lipo-CAZ-UA-Chi, as well as that of Lipo-CAZ-Chi + Lipo-UA-Chi (Table 4), were evaluated over a three-month period.

Ensuring that the formulation remains stable under storage conditions is important, as it must retain its characteristics without drug leakage, oxidation, or fusion of vesicles that can form larger particles, which may influence the in vivo performance of the formulation [89]. The Lipo-CAZ-Chi, Lipo-UA-Chi, and Lipo-CAZ-UA-Chi formulations were stable in suspension for around 30 days. After this period, the formulations showed an increase in Ø and PDI, in addition to a decrease in ζ and pH. However, none of the dispersion formulations exhibited aggregate, drug precipitation, or phase separation within the 120 days of the stability study. Thus, the physicochemical properties of the formulations remained suitable for oral administration.

### 2.2. Microbiological Analyses

#### 2.2.1. Evaluation of Antibacterial Activity

The antibacterial activity (Table 5) of the chitosan-coated liposomes containing CAZ and UA, alone or combined, was assessed against *E. coli* strains known to induce colorectal cancer (ATCC 25922, NCTC 13846, and H10407). Ceftazidime exhibited an MIC of 1.95 µg/mL across all strains, while the MIC values for UA varied. Lipo-CAZ-Chi showed promising MIC values of 0.062 µg/mL for ATCC 25922 and 0.125 µg/mL for NCTC 13846 and H10407. On the other hand, Lipo-UA-Chi displayed an MIC of 62.5 µg/mL for all strains. Lipo-CAZ-UA-Chi demonstrated MIC values of 0.975 µg/mL for ATCC 25922 and H10407 and 1.95 µg/mL for NCTC 13846. Remarkably, the combined formulation (Lipo-CAZ-Chi + Lipo-UA-Chi) exhibited the most potent antibacterial activity, with lower MIC values compared to the individual formulations (0.031 + 0.488 μg/mL for *E. coli* ATCC 25922 and 0.062 + 0.976 μg/mL for *E. coli* NCTC 13846 and *E. coli* H10407), underscoring its enhanced efficacy against the tested bacterial strains.

Previous studies have investigated the activity of CAZ encapsulated in liposomes against Gram-negative bacillus strains. Torres et al. [47] demonstrated that CAZ encapsulated in conventional liposomes (Lipo-CAZ) exhibited MIC values of 8 µg/mL and 1024 µg/mL against *Pseudomonas aeruginosa* ATCC 27853 and *P. aeruginosa* SPM-1, respectively. Farzampanah et al. [49] also assessed the efficacy of these formulations against *P. aeruginosa* ATCC 27853, showing bacterial inhibition at 2 µg/mL for CAZ and 0.5 µg/mL for Lipo-CAZ. However, there are no published studies on the activity of CAZ encapsulated in Chi-coated liposomes.

Studies investigating the inhibitory potential of UA against Gram-negative bacteria are limited. Maciąg-Dorszynska et al. [90] found no inhibition of *E. coli* MG1655 at concentrations ranging from 0.5 to 5000 µg/mL. Costa-Júnior et al. [14] reported MICs of 250 to 500 µg/mL for UA against various *P. aeruginosa* isolates. The low activity of UA against these bacteria may be attributed to its low permeability through bacterial membranes. The nanoencapsulation of UA offers a promising solution, enhancing its adhesion and release within bacterial cells [91,92]. Grumezescu et al. [93] developed iron oxide nanoparticles loaded with UA, showing an MIC and MBC of 10 and 60 µg/mL against *E. coli* ATCC 25922 and *P. aeruginosa* ATCC 27853, respectively. Khan, Yu, and Kim [73] encapsulated UA in Chi nanoparticles, with MICs of 1024 µg/mL and 512 µg/mL against *E. coli* KCTC 1682 and *P. aeruginosa* KCTC 1637, respectively.

Our findings align with reports in the literature indicating a substantial decrease in MIC and MBC for antibiotics when encapsulated in liposomes compared to their free forms. Additionally, Chi-coated liposomes enhance antibacterial activity by interacting with the negatively charged carboxyl groups on bacterial cell walls. This interaction, in synergy with the encapsulated drug, impedes the transport of essential substances for bacterial survival and facilitates drug internalization, ultimately leading to bacterial death [51,94,95].

The use of combination therapy offers a promising approach to bacterial disease treatment, potentially enhancing potency and therapeutic outcomes. By exploring drug synergy, lower doses may suffice, reducing side effects [11]. This study aimed to develop two strategies based on this principle: the co-encapsulation of CAZ and UA in a single formulation (Lipo-CAZ-UA-Chi) and a combination of both formulations (Lipo-CAZ-Chi + Lipo-UA-Chi).

Co-encapsulation, while advantageous for preserving drug viability and simplifying administration, poses challenges like drug aggregation, potentially compromising delivery efficiency and release profile control [60,96,97]. Findings in the literature align with our observations: formulations encapsulating drugs may exhibit lower antibacterial activity compared to individual drugs [98,99]. For instance, co-encapsulated azithromycin and N-acetylcysteine showed higher MIC and MBC values than those of individual encapsulations against clinical isolates of *E. coli* SA057, 0.38 and 0.75 μg/mL, respectively [98]. Similarly, tobramycin formulations exhibited reduced efficacy when co-encapsulated with N-acetylcysteine (MIC: 8–32 μg/mL and MBC: 16–64 μg/mL) [99]. Nevertheless, co-encapsulation benefits include enhanced stability and reduced toxicity, warranting further in vivo studies [99].

Preventive treatment with the use of antibiotics against bacteria related to the development of cancer, such as those observed in *Helicobacter pylori* and gastric cancer, is already widely reported in the literature [100]. Considering studies such as those by Nouri et al. [101], which found the presence of pKs *E. coli* in 23% of the CRC group compared to 7.1% in the control group, corroborating with previous research by Buc et al. [102], who identified it in 39.5% of individuals with CRC and 12.9% of patients with diverticulosis, it becomes evident that the pKs island can be considered a relevant biomarker for CRC development [101,102]. Furthermore, given its role in colorectal cancer pathogenesis, the pKs island also emerges as a promising target for the use of antibiotics as a preventive strategy. In this context, these drugs are generally used after the isolation of microorganisms in biopsy samples and aim to eliminate their colonization before the development of neoplasia [103]. Therefore, based on the results found in this study, there is the possibility of using the formulations encapsulating CAZ and UA separately with the formulation co-encapsulating the two drugs and, mainly, the *pool* of the two formulations in patients who were diagnosed with genotoxic strains of *E. coli* in an attempt to prevent the development of CRC.

While it is true that high doses of antibiotics can eliminate all microflora in the gut and potentially lead to organism death, it is important to highlight that the MIC values observed in the present study are low and have a reduced potential to cause this adverse effect. However, it is crucial to consider strategies to mitigate the risks associated with a possible complete eradication of the intestinal microbiota [104]. One strategy to address this scenario would be the concomitant administration of probiotics during antibiotic treatment. Probiotics are live microorganisms that, when consumed in adequate amounts, can confer benefits to intestinal health by restoring and maintaining the balance of the microbiota. Administering probiotics can help prevent complications arising from the suppression of the intestinal microbiota, such as the overgrowth of opportunistic pathogens and dysbiosis [105].

Another strategy would be the use of complementary therapies to restore the intestinal microbiota after the completion of antibiotic treatment. This may include the use of prebiotics, which are non-digestible food substrates that promote the growth and activity of beneficial bacteria in the gut, and fecal transplantation, a technique that involves transferring stool from a healthy donor to the patient’s intestine, aiming to restore diversity and balance in the microbiota [106,107].

#### 2.2.2. Evaluation of Antibiofilm Activity

##### Assessment of Biofilm Inhibition

**Figure 12:**
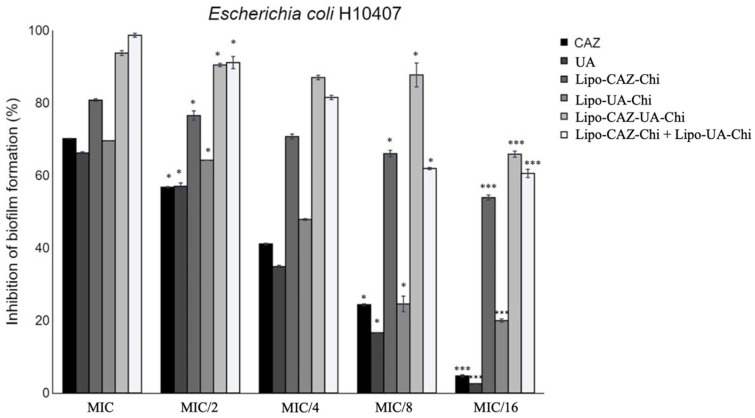
Inhibition of biofilm formation of *E. coli* H10407 strains after treatment with CAZ and UA encapsulated in Chi-coated liposomes. MIC: minimum inhibitory concentration; CAZ: ceftazidime; UA: usnic acid; Lipo-CAZ-Chi: chitosan-coated liposomes containing encapsulated CAZ; Lipo-UA-Chi: chitosan-coated liposomes containing encapsulated UA; Lipo-CAZ-UA-Chi: chitosan-coated liposomes containing co-encapsulated CAZ and UA; Lipo-CAZ-Chi + Lipo-UA-Chi: sample *pool*. * *p* < 0.05; *** *p* < 0.001 (one-way ANOVA followed by Dunnet’s post-test).

The inhibition of biofilm formation by ceftazidime CAZ, UA, Lipo-CAZ-Chi, Lipo-UA-Chi, and the combined formulation Lipo-CAZ-Chi + Lipo-UA-Chi occurred in a dose-dependent manner, with stronger inhibition observed at the MIC and reduced inhibition at the MIC/16 (Figure 12). Consequently, biofilm inhibition ranged between 4.4% and 70.1% for CAZ, 2.5% and 66.5% for UA, 54.6% and 81.1% for Lipo-CAZ-Chi, 20.4% and 69.6% for Lipo-UA-Chi, 66.7% and 94.4% for Lipo-CAZ-UA-Chi, and 61.7% and 98.1% for Lipo-CAZ-Chi + Lipo-UA-Chi. The encapsulation of CAZ and UA within the Chi-coated liposomes resulted in lower MBIC values compared to those of the free drugs, with these inhibition values being up to 13 times higher.

Knowing that the formation of biofilm provides an environment conducive to bacterial development and, therefore, causes resistance to antibiotics, it is necessary to develop alternatives that may be capable of inhibiting and eradicating it. Studies indicate that liposomes can adsorb on the surface of the bacterial biofilm and, due to this, allow for the delivery of encapsulated drugs into bacterial cells [108,109].

CAZ and UA encapsulated in Chi-coated liposomes showed lower BMIC values when compared to free drugs, with inhibition values up to 13 times higher. It was also possible to observe the inhibition potential of the biofilm formation of formulations co-encapsulating CAZ and UA, in addition to the *pool*, showing, in both cases, antibiofilm potential superior to that of free and separately encapsulated drugs. As expected, Lipo-Chi did not inhibit biofilm formation at any of the tested concentrations.

Biofilms are formed by an exopolysaccharide matrix that isolates bacteria from antibacterial substances. In this sense, effective substances for antibiofilm purposes must be able to penetrate the biofilm and eliminate bacteria [109]. Based on this, the biofilm inhibition activity exhibited by the formulations developed in the present study occurs through the action of the encapsulated CAZ and UA since both have antibiofilm potential, especially in the stages of adhesion, proliferation, and growth of biofilms [16,110].

Thus, to inhibit the formation of biofilms, there are three strategies: directly reaching bacterial cells, blocking bacterial adhesion to a surface, or interrupting communication between cells—also called *Quorum sensing*. Thus, drugs that can inhibit any of these strategies can be promising as molecules to be encapsulated in nanoformulations as antibiofilm proposals, since these make it difficult to maintain the three-dimensional structure of the biofilm [111].

To evaluate the inhibition of biofilm formation by nanosystems, some studies have been conducted. Thyagarajan et al. [112] evaluated ceftazidime encapsulated in nanoparticles coated with Chi against *E. coli* and obtained inhibition results of up to 80% at a concentration of 100 µg/mL, and they indicated that this activity was mediated by reducing the amount of total carbohydrates and proteins in the matrix surrounding the biofilms, a factor that weakens the biofilm structure and thus facilitates the entry of CAZ.

An evaluation of UA encapsulated in nanosystems for the antibiofilm elimination of Gram-negative bacteria is not described in the literature; however, this has been previously reported for Gram-positive bacteria. Francolini et al. [15] investigated the antibiofilm potential of glycosylated liposomes containing UA against biofilms of *Staphylococcus epidermidis* ATCC 35984, finding a BMIC of 16 μg/mL. For the authors, the presence of a positive charge and the sugar residues present on the liposomal surface are fundamental for promoting the interaction of liposomes with microorganisms.

In addition to drug characteristics, those related to liposomes can improve the antibiofilm potential. The literature reports that liposomes should have diameters between 100 and 300 nm to improve the penetration and targeting of antibacterial agents in a biofilm [73]. Thus, the liposomes developed in this study showed appropriate sizes for this penetration (116 to 240 nm). In addition, the presence of Chi as a coating confers a positive surface charge on the formulations of Lipo-CAZ-Chi, Lipo-UA-Chi, Lipo-CAZ-UA-Chi, and Lipo-CAZ-Chi + Lipo-UA-Chi, a factor which enables electrostatic interaction with the surface of negatively charged bacteria, which may reduce bacterial adhesion to enterocytes, resulting in the impediment of biofilm formation [110].

##### Evaluation of Preformed Biofilm Inhibition

**Figure 13:**
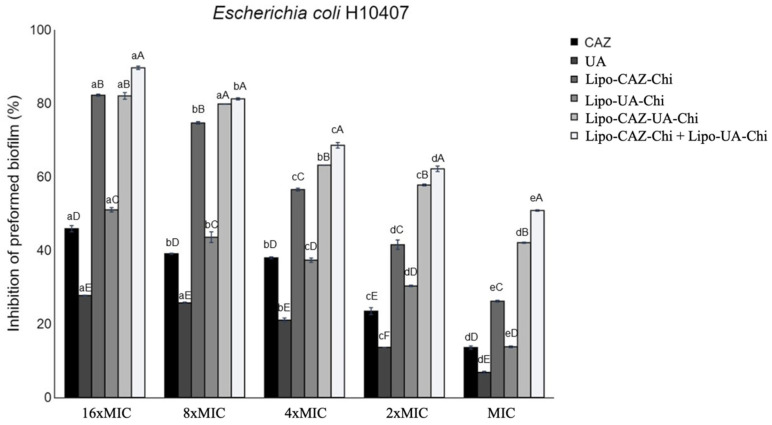
Inhibition of preformed biofilm by *E. coli* H10407 strains after treatment with CAZ and UA encapsulated in Chi-coated liposomes. MIC: minimum inhibitory concentration; CAZ: ceftazidime; UA: usnic acid; Lipo-CAZ-Chi: chitosan-coated liposomes containing encapsulated CAZ; Lipo-UA-Chi: chitosan-coated liposomes containing encapsulated UA; Lipo-CAZ-UA-Chi: chitosan-coated liposomes containing co-encapsulated CAZ and UA; Lipo-CAZ-Chi + Lipo-UA-Chi: sample *pool*. The different letters in the superscripts represent significant differences in the results of the Tukey test (*p* < 0.05).

The inhibition of preformed biofilm upon exposure to CAZ, UA, Lipo-CAZ-Chi, Lipo-UA-Chi, and Lipo-CAZ-Chi + Lipo-UA-Chi was also dose-dependent, with the highest percentage of inhibition observed at 16×MIC. Consequently, the inhibition percentages ranged between 13.1% and 46.7% for CAZ, 6.5% and 27.8% for UA, 26.4% and 82.5% for Lipo-CAZ-Chi, 13.5% and 50.4% for Lipo-UA-Chi, 42.2% and 81.1% for Lipo-CAZ-UA-Chi, and 50.6% and 89.2% for Lipo-CAZ-Chi + Lipo-UA-Chi (Figure 13).

The inhibition of the preformed biofilm consists of a direct fight against the already established biofilms through mechanisms that degrade the exopolysaccharide matrix. In this context, in addition to the elimination of bacterial cells in the biofilm, the matrix must be eradicated so that new biofilms cannot be formed [113]. In line with our results, Wan et al. [114] developed a silver nanoparticle conjugated with alginate and CAZ (Ag@MON-CE) and evaluated the inhibition of the biofilm formed by *P. aeruginosa* PA 01, which varied between 25 and 26% for free CAZ and 70 and 73 or 71% for Ag@MON-CE at concentrations from 0.07 to 1.72 mg/mL. According to the authors, this is explained by the synergy between silver ions, CAZ, and alginate, which degrade the matrix components, causing biofilm inhibition.

Therefore, our results indicate that Chi-coated liposomes encapsulating CAZ and UA can not only inhibit *E. coli* H10407 biofilm formation but also efficiently inhibit the formed biofilm, thus providing a basis for in vivo assays that can demonstrate the performance of the formulation.

## 3. Material and Methods

### 3.1. Material

#### 3.1.1. Drugs and Reagents

CAZ pentahydrate, UA, low-molecular-weight chitosan (Chi), and cholesterol were purchased from Sigma-Aldrich Chemical Co. Ltd. (St. Louis, MO, USA). Tween 80 was purchased from Neon Commercial (Suzano, SP, Brazil), and glacial acetic acid was purchased from Química Moderna (Barueri, SP, Brazil). Methanol and chloroform (HPLC grade) were purchased from Merck Millipore (Darmstadt, Germany). Phospholipids, Lipoid S100, were purchased from Lipoid KG, Germany. All other chemicals used were analytical grade.

#### 3.1.2. Bacteria

*E. coli* ATCC 25922 and *E. coli* H10407 were purchased from the American Type Culture Collection, and *E. coli* NCTC 13846 was purchased from Thermo Fisher Scientific (Waltham, MA, USA).

### 3.2. Methods

#### 3.2.1. Preparation of Chitosan-Coated Liposomes Containing CAZ, UA, and CAZ+UA

Chitosan-containing liposomes (Lipo-Chi) were prepared by the lipid film hydration method, followed by sonication. The cholesterol (CH), phosphatidylcholine (PC), and Tween 80 (7:2:1) were solubilized in an organic solvent (chloroform/methanol 9:1) under magnetic stirring. Then, the lipid film was formed by evaporating the solvent under reduced pressure. The formed lipid film was resuspended in pH 7.4 phosphate buffer, spontaneously forming large multilamellar liposomes (MLVs), which were subsequently sonicated to obtain small unilamellar liposomes (SUVs) [115]. For Chi coating on liposomes, Chi (0.5%) was solubilized in glacial acetic acid. The Chi solution was subjected to constant stirring overnight. The liposomes were added drop by drop into the Chi solution and kept under magnetic stirring for 1 h [51].

Similarly, the Chi-coated liposome containing CAZ (Lipo-CAZ-Chi) was prepared following the same steps as the Lipo-Chi preparation; however, in the hydration step of the formed film, the liposome was resuspended with the CAZ solution (2 mg/mL) solubilized in phosphate buffer at pH 7.4. As for the Chi-coated liposome containing UA (Lipo-UA-Chi), the addition of UA was performed in the solubilization step with an organic solvent at a concentration of 2 mg/mL. Finally, the formulations co-encapsulating CAZ and UA (Lipo-CAZ-UA-Chi) were made by joining the previously mentioned steps, with the solubilization of the UA (1 mg/mL) in dimethylsulfoxide (DMSO) and hydration of the film formed with the CAZ solution (1 mg/mL).

#### 3.2.2. Characterization of the Liposomes 

##### Particle Size, Polydispersity Index, Zeta Potential, and pH of the Liposomes

Lipo-Chi, Lipo-CAZ-Chi, Lipo-UA-Chi, and Lipo-CAZ-UA-Chi were subjected to physicochemical characterization through an analysis of particle size (Ø), polydispersion index (PDI), zeta potential (ζ), and pH, as previously described by Cavalcanti et al. [115]. Liposome dispersions were measured by photon correlation spectroscopy using the Zetasizer Nano-ZS90 (Malvern, Worcestershire, UK). For Ø and PDI analysis, 50 µL of liposomal dispersion was diluted in 950 µL of purified water. Measurements were performed at 25 °C with a fixed angle of 90°, and the results were expressed as the mean hydrodynamic diameter of the liposomes (nm).

The ζ of the liposomes was also measured after diluting 50 µL of the liposome dispersion in 950 µL of an ultrapure water solution. Liposome surface charge (mV) was assessed using the Zetasizer Nano-ZS90 (Malvern, Worcestershire, UK). The liposomes’ pH was measured with a glass electrode and an MS Tecnopon digital pH meter (mPA-210P, São Paulo, Brazil) at room temperature.

##### Determination of the Content and Encapsulation Efficiency of CAZ in Liposomes

To determine the CAZ content in the formulations, 25 µL of Lipo-CAZ-Chi was diluted in a 70% CH_3_OH solution (this concentration was defined based on the solubility of CAZ) and centrifuged for 10 min, and the supernatant was measured by spectrophotometry at a wavelength of 255 nm. The results were expressed as the percentage of the average absorbance. The experiment was carried out in triplicate in three independent experiments.

The encapsulation efficiency (%EE) of CAZ in the liposomes was determined by the ultrafiltration/ultracentrifugation technique, using filtration units (Amicon Ultra Centrifugal Filters; Millipore, Billerica, MA, USA). The liposome samples (400 µL) were inserted into the filters and subjected to ultracentrifugation at 8000 rpm and 4 °C for 1 h. To determine the %EE of CAZ, an aliquot of the filtered sample (25 µL) was diluted in a 70% CH_3_OH solution and measured by spectrophotometry, with the results expressed as a percentage of the average absorbance [115]. The CAZ %EE data were calculated using the equation described below:

%EE = CAZ Total − Filtered from CAZ × 100/CAZ Total

##### Determination of the Content and Efficiency of the Encapsulation of UA in Liposomes

To determine the UA content of the formulations, 20 µL of Lipo-UA-Chi was diluted in 100% CH_3_OH (this concentration was defined based on the solubility of UA), and centrifuged for 10 min, and the supernatant was measured by spectrophotometry at a wavelength of 280 nm. The results were expressed as a percentage of the average absorbance. The experiment was carried out in triplicate in three independent experiments. The %EE of UA in the liposomes was also determined by the ultrafiltration/ultracentrifugation technique, as described above.

##### Fourier-Transform Infrared Spectroscopy and X-ray Diffraction of Liposomes

For the Fourier-transform infrared spectroscopy (FTIR) analysis, the FTIR spectra of Lipo-CAZ-Chi, Lipo-UA-Chi, Lipo-CAZ-UA-Chi, Lipo-CAZ-Chi + Lipo-UA-Chi, Lipo-Chi, Lipo-NChi, Lipo-UA, Lipo-CAZ, and UA were obtained from the mixture of lyophilized samples in potassium bromide powder (KBr) and measured with a Fourier-transform spectrophotometer (Frontier). The samples were analyzed from 4000 to 400 cm^−1^, obtaining spectra with a resolution of 4 cm^−1^ [116].

The X-ray diffraction analysis of Lipo-CAZ-Chi, Lipo-UA-Chi, Lipo-CAZ-UA-Chi, Lipo-CAZ-Chi + Lipo-UA-Chi, Lipo-Chi, Lipo-NChi, Lipo- UA, Lipo-CAZ, and UA was carried out using an X-ray diffractometer (Rigaku, model Miniflex). The data were collected over an angular range of 5° to 50° 2-theta in continuous mode, using a step size of 0.02° 2-theta and a step time of 5 s [117].

##### Thermal Analysis by Thermogravimetry and Differential Scanning Calorimetry of the Liposomes

The TGA curves of Lipo-CAZ-Chi, Lipo-UA-Chi, Lipo-CAZ-UA-Chi, Lipo-CAZ-Chi + Lipo-UA-Chi, Lipo-Chi, Lipo-NChi, Lipo-UA, Lipo-CAZ, and UA were obtained using TGA equipment (TGA Q500; TA Instruments, New Castle, DE, USA). The samples (5 mg) were heated in a platinum tray at a rate of 10 °C/min, with a synthetic air flow of 20 mL/min from 0 to 600 °C [118].

The DSC curves of Lipo-CAZ-Chi, Lipo-UA-Chi, Lipo-CAZ-UA-Chi, Lipo-CAZ-Chi + Lipo-UA-CHI, Lipo-Chi, Lipo-NChi, Lipo-UA, Lipo-CAZ, and UA were obtained using a DSC (DSC Q10; TA Instruments, USA). Each analytical sample (5 mg, dry basis) was accurately weighed in an aluminum tray, and then the tray was sealed. The samples were heated from 0 to 300 °C at a constant heating rate of 10 °C/min and a flow rate of 20 mL/min in synthetic air [119].

#### 3.2.3. Stability of the Liposomes

##### Stability of Liposomes under Simulated Gastrointestinal pH Conditions

The stability of the liposomes was assessed in solutions of biological media with simulated gastric and intestinal pH, which were prepared according to Cavalcanti et al. [120]. Initially, 250 μL of Lipo-CAZ-Chi, Lipo-UA-Chi, Lipo-CAZ-UA-Chi, Lipo-CAZ-Chi + Lipo-UA-Chi, or Lipo-Chi was added to 750 μL of gastric (pH 1.2) or intestinal (pH 6.8) solution in microtubes. The microtubes were continually shook (70 rpm) at 37 °C for 2 h. At 30 min intervals until reaching 120 min, aliquots were removed and evaluated for Ø, PDI, and ζ [121].

##### Stability of the Liposomal Dispersions

The physicochemical stability of the liposomal dispersions of Lipo-Chi, Lipo-CAZ-Chi, Lipo-UA-Chi, Lipo-CAZ-UA-Chi, and Lipo-CAZ-Chi + Lipo-UA-Chi was evaluated 24 h after their development, and they were stored in a refrigerator at 4 °C. The formulations were monitored 7, 15, 30, 60, and 120 days after being formulated by evaluating the following parameters: macroscopic appearance, Ø, PDI, ζ, pH, content, and EE% for liposomes containing the drug [121].

### 3.3. Evaluation of Antibacterial Activity

The in vitro antibacterial activity of CAZ, UA, Lipo-Chi, Lipo-CAZ-Chi, Lipo-UA-Chi, Lipo-CAZ-UA-Chi, and Lipo-CAZ-Chi + Lipo-UA-Chi was evaluated using the broth microdilution method according to the Clinical and Laboratory Standards Institute [122].

Initially, Müeller–Hinton broth was poured into each well of the plates. Next, CAZ, UA, Lipo-Chi, Lipo-CAZ-Chi, Lipo-UA-Chi, Lipo-CAZ-UA-Chi, and Lipo-CAZ-Chi + Lipo-UA-Chi were added by serial dilution, and, finally, suspensions of *E. coli* ATCC 25922, *E. coli* NCTC13846, and *E. coli* H10407 were added. The microplates were incubated at 35 °C for 24 h, and the minimum inhibitory concentration (MIC) was determined by spectrophotometry at a wavelength of 630 nm.

The minimum bactericidal concentration (MBC) was determined after the MIC results. An aliquot of the microorganisms from the wells in which there was no visible growth was inoculated onto a Müeller–Hinton agar, and the plates were incubated at 35 °C for 24 h. After this period, the MBC was determined as the lowest concentration at which there was no microbial growth [122]. The whole experiment was carried out in independent triplicates.

### 3.4. Evaluation of Antibiofilm Activity

#### 3.4.1. Determination of Biofilm Formation Inhibition Concentration

The concentration at which biofilm formation was inhibited for CAZ, UA, Lipo-Chi, Lipo-CAZ-Chi, Lipo-UA-Chi, Lipo-CAZ-UA-Chi, and Lipo-CAZ-Chi + Lipo-UA-Chi against *E. coli* H10407, a biofilm-producing strain, was determined using the crystal violet method. Initially, a mixture of tryptone soy broth (TSB) + glucose was distributed into each well of the microdilution plates. Next, CAZ, AU, Lipo-Chi, Lipo-CAZ-Chi, Lipo-AU-Chi, Lipo-CAZ-AU-Chi, and Lipo-CAZ-Chi + Lipo-AU-Chi were added by serial dilution, and then bacterial suspensions were added. The microplates were incubated at 35 °C for 24 h. After incubation, the contents of the wells were aspirated and washed with 0.9% saline solution. The plates were dried, and then the adherent bacteria were fixed with 99% methanol. After fixation, the methanol was removed, and the plates were dried again.

Subsequently, the bacteria adhering to the plates were stained with 1% crystal violet. Excess dye was removed, and each well was washed with saline solution. The results were then analyzed using spectrophotometry at 570 nm (Multiskan FC microplate photometer, Thermo Scientific, Madrid, Spain). The concentration at which biofilm formation was inhibited was determined in % [123]. The whole experiment was carried out in independent triplicates.

#### 3.4.2. Determination of Preformed Biofilm Inhibition Concentration

The preformed biofilm inhibition concentration of CAZ, UA, Lipo-Chi, Lipo-CAZ-Chi, Lipo-UA-Chi, Lipo-CAZ-UA-Chi, and Lipo-CAZ-Chi + Lipo-UA-Chi against *E. coli* H10407 was determined using the crystal violet method. Initially, the strains were adjusted to the McFarland 0.5 scale. The cell count was confirmed by spectrophotometry at 630 nm, and the bacterial suspensions were distributed on flat-bottomed microdilution plates to a final concentration of 105 CFU/mL and incubated at 35 °C for 24 h. After biofilm growth, the contents of each well were aspirated, and serial dilutions of the compounds were made in tryptone soy broth (TSB) and added to each well.

The plates were incubated again at 35 ± 2 °C for 24 h. After incubation, the contents of the wells were aspirated and washed with 0.9% saline solution. The plates were dried, and then the adherent bacteria were fixed with 99% methanol. After fixation, the methanol was removed, and the plates were dried again. Subsequently, the bacteria adhering to the plates were stained with 1% crystal violet. The excess dye was removed, and each well was washed with saline solution. The results were then analyzed using spectrophotometry at 570 nm (Multiskan FC microplate photometer, Thermo Scientific, Madrid, Spain). The MIC of the preformed biofilm was determined in % [123]. The whole experiment was carried out in independent triplicates.

## 4. Conclusions

The Chi-coated liposomes encapsulating CAZ and UA developed in this study were produced by applying a simple, low-cost technique that could be scaled up industrially. In addition, they presented characteristics compatible with the potential for oral administration in future applications in antibacterial and antibiofilm therapy in vitro and in vivo, such as having a particle size below 245 nm, a positive surface charge, a high drug encapsulation efficiency, and stability in dispersions after 3 months at different temperatures and in media simulating gastric and intestinal pHs.

CAZ and UA encapsulated alone or co-encapsulated in liposomes showed in vitro antibacterial activity against *E. coli* isolates, in addition to showing the potential inhibition of biofilm formation and bacterial inhibition in the formed biofilm. Therefore, the liposomes developed in this study represent a promising therapeutic strategy against CRC-inducing bacteria. Thus, it is expected that future results may confirm the possibility of oral administration in patients who present positive biopsies for the presence of *E. coli* pks+, thus providing a new therapeutic innovation for the treatment of CRC. However, pharmacokinetic and pharmacodynamic studies must be performed in further studies to consolidate the performance of each formulation and the effectiveness of the system in vivo.

## Figures and Tables

**Table 1:** Characterization regarding particle size, polydispersion index, zeta potential, pH, content, and encapsulation efficiency of Lipo-NChi, Lipo-CAZ-Chi, Lipo-UA-Chi, Lipo-CAZ-UA-Chi, and Lipo-CAZ-Chi + Lipo-UA-Chi.

Formulation	Ø (nm)	PDI	ζ (mV)	pH	Content (%)	%EE
Lipo-NChi	106.1 ± 12.5	0.244	−2.2 ± 1.5	7.7	-	-
Lipo-CAZ-Chi	116.5 ± 5.3	0.401	+16.4 ± 0.6	5.2	98.5 ± 0.4	51.5 ± 0.2
Lipo-UA-Chi	136.6 ± 5.1	0.465	+28 ± 0.8	5.0	98.9 ± 0.3	99.9 ± 0.1
Lipo-CAZ-UA-Chi	240.3 ± 3.5	0.411	+24.9 ± 0.1	5.0	98.3 ± 0.1 (CAZ)	45.2 ± 0.5 (CAZ)
					98.7 ± 0.6 (UA)	95.6 ± 0.3 (UA)
Lipo-CAZ-Chi + Lipo-UA-Chi	212.8 ± 0.7	0.563	+22.9 ± 1.2	5.2	98.2 ± 0.2 (CAZ)	50.7 ± 3 (CAZ)
					98.5 ± 0.4% (UA)	98.9 ± 0.5 (UA)

Ø: particle size; PDI: polydispersity index; ζ: zeta potential; %EE: encapsulation efficiency; Lipo-NChi: non-chitosan-coated liposomes; Lipo-CAZ-Chi: chitosan-coated liposomes containing encapsulated CAZ; Lipo-UA-Chi: chitosan-coated liposomes containing encapsulated UA; Lipo-CAZ-UA-Chi: chitosan-coated liposomes containing co-encapsulated CAZ and UA; Lipo-CAZ-Chi + Lipo-UA-Chi: sample pool.

**Table 2:** Long-term stability of chitosan-coated liposome containing ceftazidime.

Months			
Parameters	1	2	3
Ø (nm)	118.4 ± 4.3	159.9 ± 7.1	201.8 ± 2.0
PDI	0.412	0.612	0.612
ζ (mV)	+17.6 ± 1.0	+12.5 ± 3.5	+9.1 ± 2.3
pH	5.1	4.5	4.5
CAZ content (%)	98.1 ± 0.2	95.1 ± 0.9	95.4 ± 1.0
CAZ %EE	50.9 ± 0.5	47.4 ± 0.2	40.2 ± 0.7

Ø: particle size; PDI: polydispersity index; ζ: zeta potential; CAZ: ceftazidime.

**Table 3:** Long-term stability of chitosan-coated liposome containing usnic acid.

Months			
Parameters	1	2	3
Ø (nm)	141.3 ± 4.3	289.9 ± 7.1	303.5 ± 6.4
PDI	0.456	0.636	0.726
ζ (mV)	+24.6 ± 2.2	+15.8 ± 4.5	+7.8 ± 3.1
AU content (%)	95.2 ± 1.2	94.2 ± 1.5	92.7 ± 0.2
AU %EE	99.9 ± 0.3	90.2 ± 1.0	80.1 ± 0.6

Ø: particle size; PDI: polydispersity index; ζ: zeta potential; AU: usnic acid.

**Table 4:** Long-term stability of chitosan-coated liposome containing ceftazidime and usnic acid.

Months			
Lipo-CAZ-AU-Chi			
Parameters	1	2	3
Ø (nm)	235.2 ± 7.4	256.4 ± 2.4	345.1 ± 2.0
PDI	0.419	0.471	0.526
ζ (mV)	+19.1 ± 4.3	+17.1 ± 2.2	+9.1 ± 2.0
pH	5.0	4.9	4.7
CAZ content (%)	98.1 ± 0.5	95.8 ± 0.1	92.6 ± 0.3
CAZ %EE	45.1 ± 0.2	42.4 ± 1.0	38.4 ± 0.6
AU content (%)	96.9 ± 0.5	91.2 ± 0.4	91.6 ± 0.3
AU %EE	94.9 ± 0.4	79.2 ± 0.1	48.4 ± 0.6

**Lipo-CAZ-Chi + Lipo-AU-Chi**			
**Parameters**	**1**	**2**	**3**
Ø (nm)	232.4 ± 2.5	91.5 ± 0.1	326.2 ± 2.7
PDI	0.632	0.701	0.821
ζ (mV)	+19.3 ± 1.6	+7.1 ± 0.6	+4.1 ± 1.0
pH	5.0	4.8	3.6
CAZ content (%)	91.2 ± 0.6	90.6 ± 1.0	92.4 ± 2.6
CAZ %EE	45.6 ± 2.1	30.1 ± 2.0	25.1 ± 0.2
AU content (%)	97.5 ± 1.0	96.4 ± 0.2	94.9 ± 0.9
AU %EE	91.5 ± 0.1	68.1 ± 3.0	54.6 ± 1.2

Ø: particle size; PDI: polydispersity index; ζ: zeta potential; CAZ: ceftazidime; AU: usnic acid.

**Table 5:** Evaluation of the antibacterial activity of CAZ, UA, Lipo-CAZ-Chi, Lipo-UA-Chi, Lipo-CAZ-UA-Chi, and Lipo-CAZ-Chi + Lipo-UA-Chi against *E. coli* ATCC 25922, *E. coli* NCTC 13846, and *E. coli* H10407.

Bacteria	CAZ		UA		Lipo-CAZ-Chi		Lipo-UA-Chi		Lipo-CAZ-UA-Chi		Lipo-CAZ-Chi + Lipo-UA-Chi	
	MIC	MBC	MIC	MBC	MIC	MBC	MIC	MBC	MIC	MBC	MIC	MBC
	µg/mL											
*Escherichia coli* ATCC 25922	1.95	1.95	500	1000	0.062	0.062	62.5	62.5	0.975/0.975	0.975/0.975	0.031/0.488	0.031/0.488
*E. coli* NCTC 13846	1.95	1.95	1000	1000	0.125	0.250	62.5	62.5	1.95/1.95	1.95/1.95	0.062/0.976	0.062/0.976
*E. coli* H10407	1.95	1.95	250	1000	0.125	0.125	62.5	62.5	0.975/0.975	0.975/0.975	0.062/0.975	0.062/0.975

CAZ: ceftazidime; UA: usnic acid; Lipo-CAZ-Chi: chitosan-coated liposomes containing encapsulated CAZ; Lipo-UA-Chi: chitosan-coated liposomes containing encapsulated UA; Lipo-CAZ-UA-Chi: chitosan-coated liposomes containing co-encapsulated CAZ and UA; Lipo-CAZ-Chi + Lipo-UA-Chi: sample pool; MIC: minimum inhibitory concentration; MBC: minimum bactericidal concentration; ATCC: American Type Culture Collection; NCTC: National Collection of Type Cultures.

## Data Availability

All data generated or analyzed during this study are included in this published article. The data are not publicly available due to confidentiality agreements with participating organizations.
